# Curcumin enhances the lung cancer chemopreventive efficacy of phospho-sulindac by improving its pharmacokinetics

**DOI:** 10.3892/ijo.2013.1995

**Published:** 2013-06-27

**Authors:** KA-WING CHENG, CHI C. WONG, GEORGE MATTHEOLABAKIS, GANG XIE, LIQUN HUANG, BASIL RIGAS

**Affiliations:** Division of Cancer Prevention, Department of Medicine, Stony Brook University, Stony Brook, NY 11794-8173, USA

**Keywords:** phospho-sulindac, curcumin, lung cancer, cellular uptake, pharmacokinetics

## Abstract

Phospho-sulindac (PS) is a safe sulindac derivative with promising anticancer efficacy in colon cancer. We evaluated whether its combination with curcumin could enhance the efficacy in the treatment of lung cancer. Curcumin, the principal bioactive component in turmeric, has demonstrated versatile capabilities to modify the therapeutic efficacy of a wide range of anticancer agents. Here, we evaluated the effect of co-administration of curcumin on the anticancer activity of PS in a mouse xenograft model of human lung cancer. Curcumin enhanced the cellular uptake of PS in human lung and colon cancer cell lines. To assess the potential synergism between curcumin and PS *in vivo*, curcumin was suspended in 10% Tween-80 or formulated in micellar nanoparticles and given to mice by oral gavage prior to the administration of PS. Both formulations of curcumin significantly improved the pharmacokinetic profiles of PS, with the 10% Tween-80 suspension being much more effective than the nanoparticle formation. However, curcumin did not exhibit any significant modification of the metabolite profile of PS. Furthermore, in a mouse subcutaneous xenograft model of human lung cancer, PS (200 mg/kg) in combination with curcumin (500 mg/kg) suspended in 10% Tween-80 (51% inhibition, p<0.05) was significantly more efficacious than PS plus micelle curcumin (30%) or PS (25%) or curcumin alone (no effect). Consistent with the improved pharmacokinetics, the combination treatment group had higher levels of PS and its metabolites in the xenografts compared to PS alone. Our results show that curcumin substantially improves the pharmacokinetics of PS leading to synergistic inhibition of the growth of human lung cancer xenografts, representing a promising drug combination.

## Introduction

Lung cancer is the leading cause of cancer-related deaths worldwide. Despite advances in early detection and chemotherapy, its prognosis is generally poor, with a 5-year survival of approximately 15%. Given a lack of effective therapeutics, the use of chemopreventive agents that abrogate lung carcinogenesis represents a promising approach for controlling this disease.

Compelling evidence has emerged that non-steroidal anti-inflammatory drugs (NSAIDs) can reduce the incidence of various cancers and limit metastatic disease (1–3). However, the chronic use of NSAIDs is associated with significant gastrointestinal and renal toxicities. To reduce toxicity and enhance the efficacy of conventional NSAIDs, our group has developed novel phospho-derivatives of NSAIDs. One such derivative is phospho-sulindac (PS, OXT-328), which is efficacious in the prevention and treatment of colon and breast cancer in preclinical models (4–7) and shows a favorable safety profile (5). In contrast, PS as a single agent was ineffective in the treatment of human lung cancer xenografts (8), which prompted us to develop more potent PS-based therapy incorporating other anticancer agents. Here, we describe the combination of PS with curcumin for the prevention of lung cancer.

Curcumin, the principal bioactive component in turmeric, exhibits antitumorigenic activities (9–11). In pre-clinical models of lung cancer, however, curcumin as a single agent has demonstrated poor efficacy (<30%) (12); and according to one report, it may even promote *Kras*-driven lung tumorigenesis in mice (13). On the other hand, curcumin significantly potentiates the antitumor activity of sulindac, docetaxel and gefitinib in animal models (14–16). Curcumin can also overcome resistance to anticancer drugs such as paclitaxel, thalidomide and bortezomib (17,18). Thus, curcumin is a versatile chemosensitizer for mechanistically diverse anticancer agents.

Here, we demonstrate that curcumin potentiates the anticancer efficacy of PS in human non-small cell lung cancer (NSCLC) cells and that such a combination synergistically inhibits the growth of A549 xenografts in mice. These findings suggest that PS plus curcumin is a promising combination for the prevention of NSCLC.

## Materials and methods

### Reagents

Phospho-sulindac (OXT-328) was a gift from Medicon Pharmaceuticals, Inc., Setauket, NY, USA. Cell culture reagents were purchased from Cellgro (Herndon, VA, USA). Other reagents, unless otherwise stated, were obtained from Sigma-Aldrich (St. Louis, MO, USA).

### Cell culture

Human NSCLC (A549), breast (MCF-7 and MDA-MB-231), colon (SW480) and pancreatic (MIAPaCa-2) cancer cell lines were obtained from American Type Culture Collection (ATCC) and maintained in the recommended culture media containing 10% fetal bovine serum and penicillin/streptomycin. All experiments were performed with cells between passages 1 and 10.

### Curcumin formulation

Polymeric nanoparticles of poly(ɛ-caprolactone) (11,000)-polyethylene glycol (5,000) with entrapped curcumin were prepared according to the nanoprecipitation-solvent displacement method (19,20). Briefly, 50 mg of polymer and 5 mg of curcumin were dissolved in 1 ml of acetone and the solution was added dropwise to 2 ml of water under constant stirring. The organic solvent was allowed to slowly evaporate under reduced pressure and the resulting suspension was centrifuged to remove aggregates and drug precipitates. Curcumin concentration was determined using HPLC. Ten minutes after diluting the suspension in water, we determined the size and zeta potential of the nanoparticles using Dynamic Light Scattering (Zeta-Plus Brookhaven instrument, Holtsville, NY, USA). Particle size was also determined using transmission electron microscopy. Curcumin loading in the nanoparticles was 8±0.3%. The mean nanoparticle size was 45.2 nm and their polydispersity index was 0.271±0.005.

### Cell growth inhibition assays

Cell viability was determined by a modified colorimetric assay using 3-[4,5-dimethylthiazol-2-yl]-2,5-diphenyltetrazolium bromide (MTT). Briefly, A549 cells seeded in 96-well plates were treated with different concentrations of PS for 24 h with or without pretreatment with curcumin for 3 h. The culture medium was removed and replaced with 100 μl complete medium containing 0.5 mg/ml MTT. Following 4-h incubation at 37°C, 100 μl of a solution containing 10% SDS and 0.01 N HCl was added. The plate was incubated and gently mixed until MTT formazan crystals were dissolved. Absorbance at 570 nm was measured on a microplate reader and IC_50_ was calculated after subtraction of blank values.

### Apoptotic cell death analysis

After drug treatment, cells were trypsinized, washed once with PBS and stained with Annexin V/propidium iodide. Percentage of apoptotic cells was determined by flow cytometry on a FACSCalibur.

### Cellular uptake assay

For the uptake study, cells were seeded into 12-well plates at a cell density of 2.5×10^5^ per well. After overnight incubation, uptake experiments were carried out using serum-free media. After washing the monolayer twice, the cells were pre-incubated for 15 min with serum-free media with or without transporter inhibitors. PS was then added and incubated for 1 h. At the end of the incubation, the medium was quickly aspirated. The cells were washed twice, each with 2 ml of ice-cold transport buffer containing 0.2% bovine serum albumin (BSA). Finally, the cells were rinsed with 0.5 ml of ice-cold transport buffer without BSA. Cells were collected with 0.5 ml of 50% methanol. Extraction was performed by sonication for 5 min followed by the addition of 0.5 ml of ice-cold methanol and centrifuged at 17,000 × g for 12 min. The supernatant was collected and analyzed by HPLC. The protein pellet was re-dissolved in 0.1 N NaOH and the protein content was determined by the Bradford assay. All the uptake values were corrected against protein content.

### Pharmacokinetic studies in mice

This and subsequent animal studies were approved by the Institutional Animal Care and Use Committee (IACUC) of Stony Brook University. Mice (n=2) were given a single dose of the following treatments: i) PS 200 mg/kg; ii) PS 200 mg/kg plus curcumin 500 mg/kg, in 10% Tween-80; and iii) PS 200 mg/kg plus micellar curcumin 500 mg/kg. At designated time-points, mice were euthanized by CO_2_ inhalation and blood was collected and immediately centrifuged. The resulting plasma was deproteinized by immediately mixing it with 2.5× volumes of acetonitrile. The deproteinized samples were analysed by HPLC as described below.

### A549 xenografts

Female nude mice 6–7 weeks old were purchased from Harlan Sprague-Dawley, Indianapolis, IN, USA. At 7–8 weeks of age, four groups of mice (n=6 per group) were pre-treated for 3 days with: i) vehicle; ii) PS 200 mg/kg/d; iii) curcumin 500 mg/kg/d; and iv) PS 200 mg/kg/d plus curcumin 500 mg/kg/d. Then, the mice were inoculated subcutaneously into both flanks with A459 cells (2×10^6^ each) suspended in 100 μl complete F12K medium: Matrigel Matrix gel (BD Biosciences, San Jose, CA, USA) (1:1, v/v). The treatment was resumed one day after tumor implantation and continued daily until the end of the study. The tumors were measured twice a week with a digital microcaliper and tumor volumes were calculated using the formula: tumor volume = [length × width × (length + width/2) × 0.56]. At the end of the experiment, the animals were sacrificed and their tumors were removed. The levels of PS and its metabolites in the tumors were determined by HPLC (21).

### HPLC analysis

The HPLC system consisted of a Waters Alliance 2695 Separations Module equipped with a Waters 2998 photo-diode array detector (Waters, Milford, MA, USA) and a Thermo BDS Hypersil C18 column (150×4.6 mm, particle size 3 μm) (Thermo Fisher Scientific, Waltham, MA, USA). The mobile phase consisted of a gradient between solvent A [(trifluoroacetic acid, acetonitrile, H_2_O (0.1:4.9:95, v/v/v)] and 100% acetonitrile.

### Statistical analyses

Data are expressed as mean ± SEM. Statistical analyses were performed by ANOVA. P-values <0.05 were considered statistically significant.

## Results

### Curcumin synergizes with PS in inhibiting the growth of lung cancer cells in vitro

**Figure 1:**
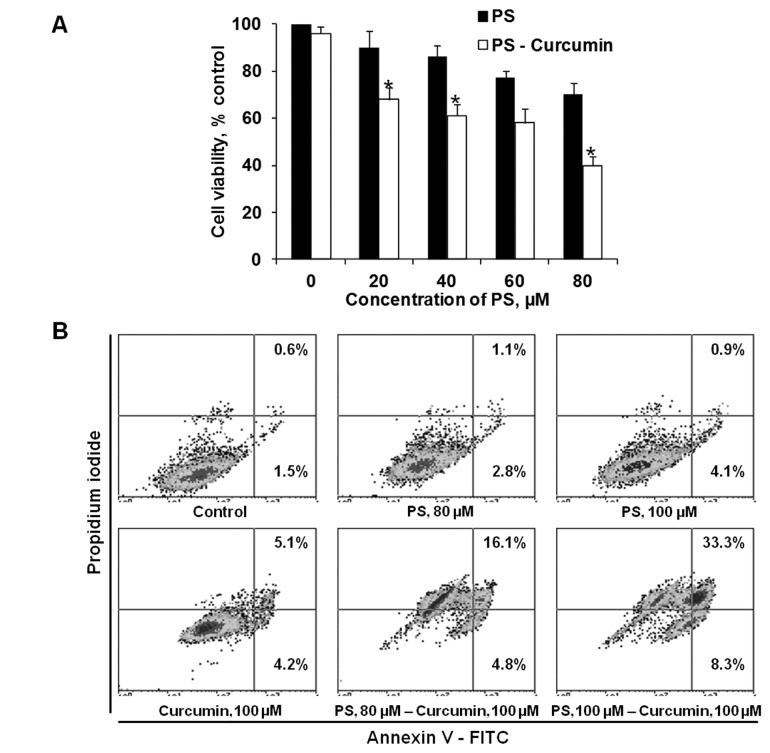
PS and curcumin synergistically inhibit the growth of A549 lung cancer cells *in vitro*. (A) Percent cell viability of A549 cells following treatment with PS alone (20–80 μM) or in combination with curcumin (100 μM) pretreatment. ^*^p<0.05 compared to control. (B) A549 cells were treated with PS (80 or 100 μM) alone or in combination with curcumin (100 μM). Cells were stained with Annexin V/PI and analyzed by flow cytometry and the percentages of cell populations are indicated in each quadrant. Annexin V (+) cells are apoptotic.

Pretreatment with curcumin sensitizes A549 lung cancer cells to the cytotoxic effect of PS. As shown in Fig. 1A, pretreatment of A549 cells with non-cytotoxic levels of curcumin 100 μM enhanced the cytotoxicity of PS. Following treatment with 100 μM curcumin, reductions of cell viability were as follows: PS 80 μM alone, 30%; curcumin alone, 4%; and PS plus curcumin, 60%. A similar synergistic effect was also observed in the induction of apoptosis. After 20-h incubation, the percentage of apoptotic cells treated with 80 and 100 μM PS with curcumin 100 μM was 20.9 and 41.6%, respectively, compared to 3.9 and 5.0% with 80 and 100 μM PS alone. These findings establish that curcumin potentiates the cytotoxic activity of PS in lung cancer cells.

### Curcumin enhances the cellular uptake of PS in cancer cells

Since curcumin is known to synergize with other compounds and to also inhibit cellular efflux transporters, we reasoned that curcumin might have synergistic activity with PS through an effect on ATP-binding cassette (ABC) transporters which are implicated in the cellular efflux of xenobiotics, such as anticancer drugs. Isoform-specific inhibitors of ABC transporters enable better understanding of the role of individual ABC transporter(s) in the efflux of a drug (22). To identify specific ATP transporters involved in the efflux of PS, changes in the cellular levels of PS were evaluated in the presence of isoform-specific inhibitors. In this study, the involvement of efflux transporters was studied using inhibitors of multidrug resistance proteins (MRPs; MK571), breast cancer resistance protein (BCRP; Ko143) and P-glycoprotein (P-gp; verapamil).

**Figure 2:**
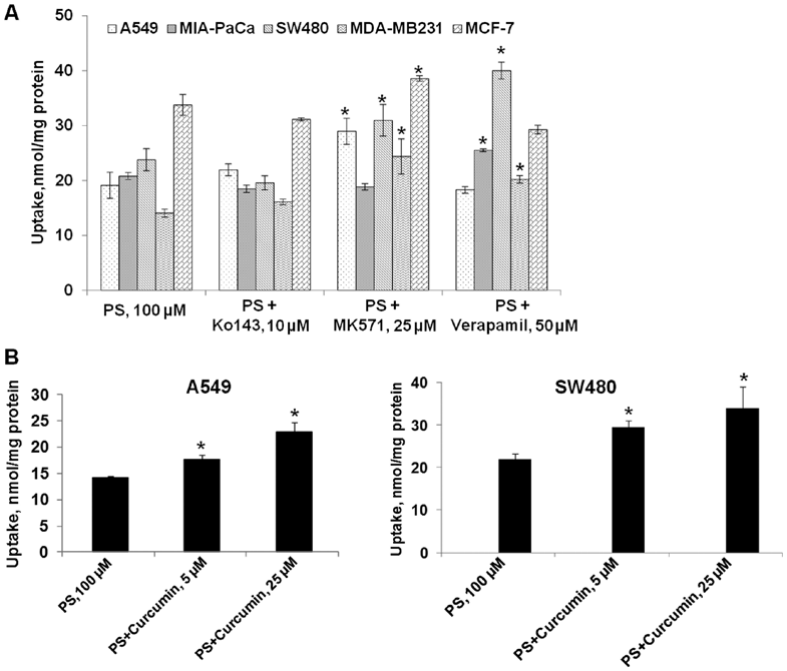
Curcumin and efflux transport inhibitors enhance the cellular uptake of PS. (A) Effect of transporter inhibitors on the cellular uptake of PS. Inhibitor of P-glycoprotein (Verapamil), multidrug resistance protein (MK571) or breast cancer resistance protein (Ko143) was incubated with 100 μM PS in five human cancer cell lines for 1 h and the intracellular PS levels were determined by HPLC. (B) Effect of curcumin on cellular uptake of PS. Curcumin (5 or 25 μM) was co-added with PS in A549 and SW480 cells for 1 h and the intracellular PS levels were determined by HPLC. Values are mean ± SEM of at least three independent experiments.

In A549 cells, co-incubation of PS with MK571 resulted in a 51% increase in the intracellular accumulation of PS (Fig. 2A). Ko143 and verapamil had no effect on the uptake of PS in A549 cells. In SW480 cells, MK571 and verapamil, but not Ko143, enhanced the cellular accumulation of PS by 30 and 67%, respectively (Fig. 2A). It appeared that multidrug resistance proteins (MRPs) are involved in PS efflux in both A549 and SW480 cells; while P-glycoprotein (P-gp) is only involved in the efflux of PS in SW480. BCRP, on the other hand, has little impact on the efflux of PS. Thus, the specific ABC transporter(s) involved in the efflux of PS is cell-line dependent.

Given that curcumin can inhibit efflux transporters including MRPs and P-gp, we explored the effect of curcumin on the uptake of PS in A549 and SW480 cells. The effect of curcumin (5 and 25 μM) on the accumulation of PS is shown in Fig. 1B. At 5 and 25 μM, curcumin increased the intracellular levels of PS in A549 cells by 23 and 60%, respectively. Similarly, curcumin also increased the uptake of PS in SW480 cells by 37% at 5 μM and 54% at 25 μM. Therefore, co-incubation with curcumin recapitulated the effect of transporter inhibitors. Since curcumin is an inhibitor of MRPs and P-gp, these findings suggest that curcumin may enhance PS accumulation via inhibition of efflux transports in these cancer cell lines.

### PS and curcumin synergistically inhibit the growth of A549 xenografts in mice

**Figure 3:**
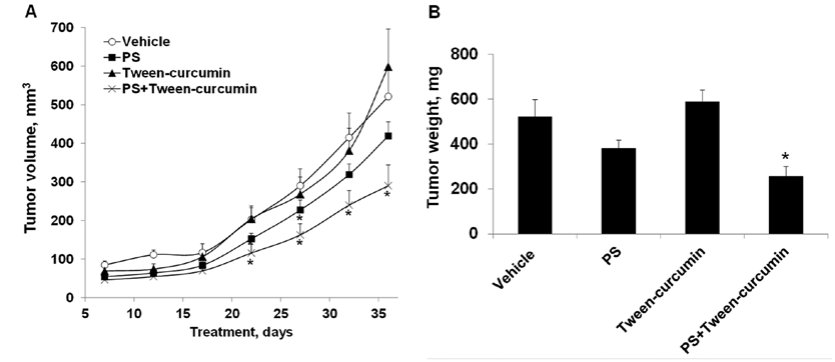
Combination of PS and curcumin synergistically inhibits the growth of A549 lung cancer xenografts in nude mice. (A) Nude mice (n=6) were pre-treated with vehicle, PS (200 mg/kg), curcumin (500 mg/kg in 10% Tween-80), or a combination of PS (200 mg/kg) and curcumin (500 mg/kg in 10% Tween-80) for 3 days, after which A549 cells were inoculated subcutaneously into both flanks. Treatment was continued until the end of the study and tumor volume was measured at designated time-points. (B) Tumor weights of the different groups were determined at end-point. Values are mean ± SEM. ^*^p<0.05 compared to control.

We investigated the antitumor efficacy of PS, curcumin or their combination in subcutaneous xenografts of A549 human lung cancer cells in nude mice. PS and curcumin, when given alone, did not significantly inhibit the growth of A549 xenografts (Fig. 3A). PS alone produced a small inhibition of tumor growth that was statistically significant on days 17–27 after tumor implantation; whereas neither formulation of curcumin was effective for the duration of the study. On the other hand, PS in combination with curcumin suspended in 10% Tween-80 synergistically inhibited the growth of A549 xenografts and the effect was statistically significant (p<0.05) beginning on day 12 until the end of the study (day 36). At the end of the study, tumor volume of each group was as follows: control, 521±76 mm^3^; PS, 419±36 mm^3^; curcumin in 10% Tween-80: 599±98 mm^3^; PS plus curcumin, 290±54 mm^3^. This corresponds to a reduction in tumor volume of 19.6 and 44.3% for PS and PS plus curcumin, respectively. In terms of tumor weight (Fig. 3B), a reduction was observed in the PS (27%, p=0.06) and the PS plus curcumin (51%, p<0.01) groups, but not in the curcumin-treated group. Of note, treatment with PS plus curcumin in 10% Tween-80 was significantly more effective than PS or curcumin alone (p<0.05). Surprisingly, no synergistic antitumor activity was observed for the combination between PS and nanoparticles-encapsulated curcumin (data no shown). Taken also into account that PS plus curcumin in 10% Tween-80 generated a better PK profile for PS than did PS plus curcumin in nanoparticles, the results of the xenograft study likely suggest the existence of a threshold level for the pharmacological effective dose of PS and/or its metabolites.

PS, curcumin or their combination produced no apparent adverse effects on the mice during the whole duration of the study; and the mean body weights of the treatment groups were comparable to that of the control. At sacrifice, the body weight of the 4 groups of mice was as follows: control, 23±2 g; PS, 23±1 g; curcumin, 23±1 g; and PS plus curcumin, 24±3 g.

### Curcumin improves the bioavailability of PS in mice

**Figure 4:**
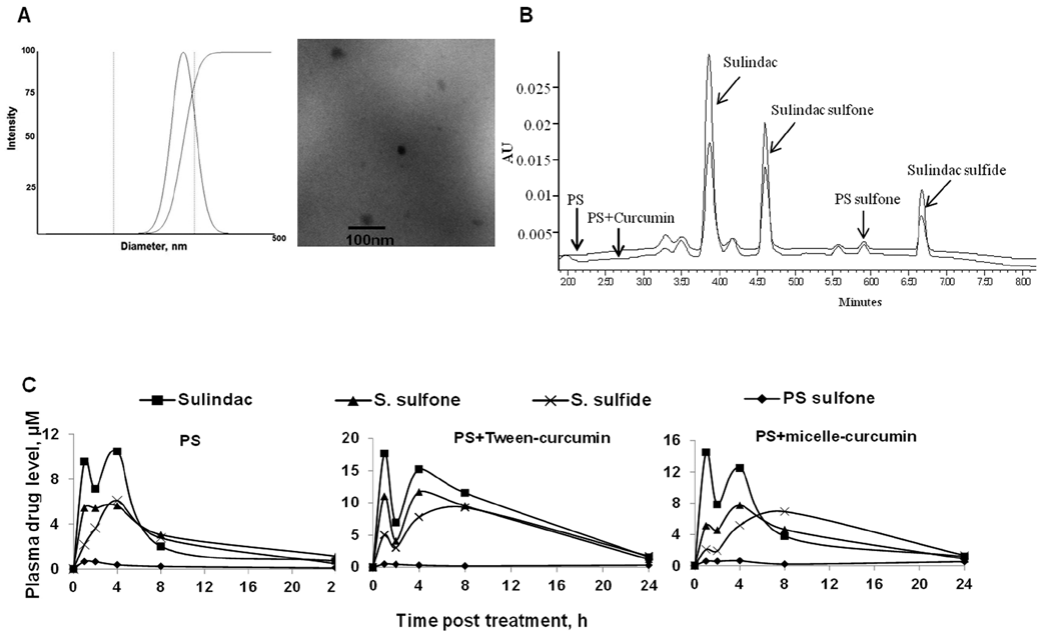
Curcumin enhances the systemic bioavailability of PS in mice. (A) The formulation of curcumin in micelle nanoparticles. The average hydrodynamic diameter (left) and the transmission electron microscopy (TEM) images (right) of curcumin-incorporated micelles. (B) Representative HPLC chromatograms (328 nm) for plasma samples from the PS- and PS-curcumin-treated mice, respectively. PS metabolites are denoted with arrows. (C) *In vivo* pharmacokinetic study in mice: PS (200 mg/kg), PS (200 mg/kg) plus curcumin (500 mg/kg in 10% Tween-80), or PS (200 mg/kg) plus curcumin (500 mg/kg in micelles) were administered to mice as a single oral dose and blood samples were collected at the indicated time-points. Plasma levels of the three main PS metabolites, sulindac, sulindac sulfone and sulindac sulfide were determined by HPLC. Values are the average of duplicate samples (all within 15% of each other).

Having shown that curcumin enhances the cellular uptake of PS and synergises with PS in inhibiting the growth of human lung cancer xenografts, we next examined the effect of curcumin co-administration on the bioavailability of PS in mice. Curcumin in two formulations, 10% Tween-80 or encapsulated in nanoparticles, was given to the mice 30 min prior to PS administration. As shown in Fig. 4 and Table I, curcumin in both formulations increased the bioavailability of PS *in vivo*. PS is rapidly metabolized *in vivo* into several metabolites, of which quantitatively most important are sulindac, sulindac sulfide and sulindac sulfone (23).

Peak plasma levels (C_max_) of sulindac, sulindac sulfone and sulindac sulfide were much higher after the co-administration of PS with curcumin. The C_max_ of sulindac, the main PS metabolite, was increased by 70% (17.7 μM) and 40% (14.5 vs. 10.4 μM for PS alone) when co-administered with curcumin in 10% Tween-80 or nanoparticles, respectively. As with the case for sulindac, the C_max_ of sulindac sulfone and sulindac sulfide were also higher following administration of PS with curcumin. Intact PS, however, was not detected in any of the three treatments, presumably due to the high carboxylesterase activity in the mouse blood (23). In terms of total plasma AUC_0–24 h_, PS given with curcumin in 10% Tween-80 (500 μM*h) or nanoparticles (309 μM*h) increased the sum of AUC_0–24 h_ of all metabolites by 2.4- and 1.5-fold, respectively, compared to PS alone (211 μM*h). Interestingly, curcumin suspended in 10% Tween-80 proved to be more effective than the nanoparticle formation in enhancing the pharmacokinetics of PS, giving rise to much higher levels (30–50%) of PS metabolites. On the other hand, curcumin had no apparent effect on the metabolism of PS in mice, as indicated by their similar metabolic profile (sulindac > sulindac sulfone = sulindac sulfide > PS sulfone). Our results suggest that the co-administration with curcumin enhances the bioavailability of PS without affecting its metabolism *in vivo* (Fig. 4B and C).

### Curcumin enhances PS levels in A549 xenografts

**Figure 5:**
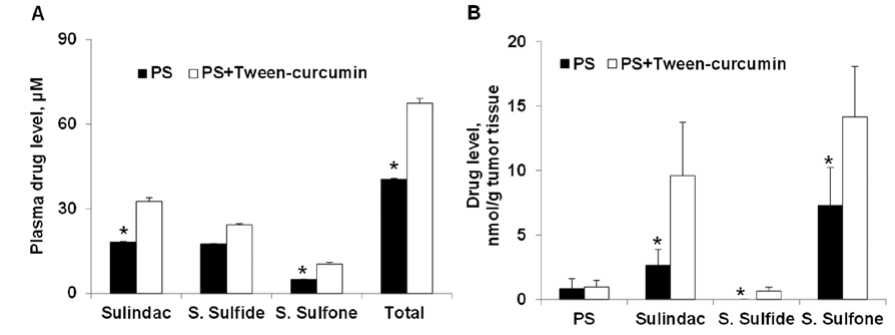
Curcumin co-administration enhances PS levels in tumors. The plasma and tumor levels of PS and its metabolites were measured at the end of the lung cancer xenograft study. (A) Plasma levels of sulindac, sulindac sulfone and sulindac sulfide. (B) Tumor levels of PS and its metabolites. Values are mean ± SEM. ^*^p<0.05 compared to control.

Given the enhanced efficacy of the combined PS and curcumin treatment, we assessed drug levels in the plasma and A549 xenografts from PS, curcumin and the combination treatment groups (Fig. 5). Compared to PS alone, PS plus curcumin generated higher levels of its three main metabolites in the blood. In the A549 xenografts, the levels of sulindac, sulindac sulfide and sulindac sulfone in the PS plus curcumin group were 1-, 3- and 5-fold higher than those of the PS alone group. The higher levels of PS metabolites are consistent with the higher efficacy achieved with the PS and curcumin combination treatment. We also detected minute levels of curcumin glucuronide in the plasma of curcumin and PS plus curcumin groups, but there was no significant difference in its levels between the two groups. Neither curcumin, nor its glucuronide, was detected in A549 xenografts.

## Discussion

Our study demonstrates that curcumin enhances the efficacy of PS against human lung cancer in pre-clinical models. We establish that curcumin: a) potentiates the cytotoxicity of PS *in vitro*; b) increases the cellular uptake of PS in cancer cells; c) improves the systemic bioavailability of PS and its metabolites; and d) enhances the delivery of PS and its metabolites to the A549 xenografts, leading to their synergistic growth inhibition by the two agents.

The combination of PS and curcumin suspended in 10% Tween-80 exerted a strong inhibitory effect on A549 xenografts in nude mice, reducing tumor volume by 44% and tumor weight by 51% (both p<0.05). In contrast, PS (20% inhibition) or curcumin (no inhibition) alone did not produce a significant inhibitory effect on A549 xenografts. A key finding of this study is that curcumin co-administration improves the bioavailability and pharmacokinetic properties of PS, as illustrated by higher peak levels (C_max_) and a 2.4-fold increase in total AUC_0–24 h_ of PS and its metabolites. Consequently, administration of PS plus curcumin resulted in much greater accumulation of PS and its metabolites in A549 xenografts compared to PS alone. The pharmacokinetic profile of curcumin, however, was not affected when combined with PS. Given that curcumin (and its metabolites) showed no detectable accumulation in tumors and had no tumor inhibitory effect, these data indicate that the synergistic effect of PS plus curcumin in 10% Tween-80 is predominantly a consequence of the enhanced delivery of PS and its metabolites to the tumors.

The chemo-sensitizing effect of curcumin is dependent upon its ability to inhibit ATP-binding cassette (ABC) proteins. ABC transporters are the primary active transporters that mediate the efflux of xenobiotics such as anticancer drugs; and their overexpression in cancer cells is associated with multidrug resistance (24). Curcumin is a promiscuous inhibitor of drug transporters from the ABC family, including P-glycoprotein (MDR1/ABCB1) (25), BCRP (ABCG2) (26) and multiple MRPs [MRP1/ABCC1, MRP2/ABCC2 (27,28), MRP5/ABCC5 (29)]. Our study revealed that curcumin enhances the bioavailability of PS *in vitro* and *in vivo* by improving the cellular uptake of PS. The uptake of PS into lung and colon cancer cells *in vitro* is influenced by drug efflux transporters, such as MRPs and P-glycoproteins, which decrease the accumulation and cellular toxicity of PS. Curcumin, at non-cytotoxic levels, antagonizes the effect of these transporters and thus increases the cellular uptake of PS in cancer cells, thereby potentiating its cytotoxic activity *in vitro*.

Curcumin may improve the bioavailability of PS in two ways. First, curcumin may inhibit efflux transporters in the intestinal barrier, thus enhancing the absorption of PS. Second, curcumin may also inhibit drug efflux in tumor xenografts, resulting in increased biodistribution of PS and its metabolites to the target tissue. The higher levels of PS and its metabolites in the A549 xenografts were consequential, as they correlated with reduced tumor volume. On the other hand, curcumin did not affect the metabolism of PS by carboxylesterases and cytochrome P450s. Our findings support the idea that curcumin potentiates the antitumor activity of PS through enhanced delivery of PS and its metabolites to tumors. The three quantitatively important metabolites of PS (sulindac, sulindac sulfide and sulindac sulfone) are known to have anticancer properties (23) both through COX-dependent and -independent pathway (30).

In conclusion, our data demonstrate that the co-administration of PS and curcumin synergistically inhibits the growth of human lung cancer xenografts in nude mice. The enhanced efficacy is attributed to inhibition of efflux transporters by curcumin, leading to improved PS bioavailability including the target tumor. This promising drug combination merits further evaluation.

## Figures and Tables

**Table I:** Pharmacokinetic parameters of major metabolites of PS following administration of a single oral dose of PS (200 mg/kg) alone or in combination with curcumin (500 mg/kg) in 10% Tween-80 (Tw-curcumin) and micelles (Mic-curcumin), respectively.

	AUC_0–24 h_			C_max_, μM			T_max_, h		
			
PS metabolite	PS	PS+Tw− curcumin	PS+Mic− curcumin	PS	PS+Tw− curcumin	PS+Mic− curcumin	PS	PS+Tw− curcumin	PS+Mic− curcumin
Sulindac	77.5	201.9	110.8	10.4	17.7	14.5	4	1	1
Sulindac sulfide	57.3	137.6	100.3	6.1	9.3	6.9	4	8	8
Sulindac sulfone	70.3	153.6	88.5	5.6	11.7	7.7	4	4	4
PS sulfone	5.8	6.8	9.5	0.66	0.49	0.63	1	1	4
